# Supplementary material for: Long Non-Coding RNA PANTR1 is Associated with Poor Prognosis and Influences Angiogenesis and Apoptosis in Clear-Cell Renal Cell Cancer
Source: Cancers (Basel). 2020 May 10;12(5):1200. doi: 10.3390/cancers12051200 (PMC7281347; doi:10.3390/cancers12051200)
Supplement: Supplementary file 1 [file cancers-12-01200-s001.pdf]

cancers

Long Non-Coding RNA PANTR1 is Associated with Poor Prognosis and Influences Angiogenesis and Apoptosis in Clear-Cell Renal Cell Cancer

Maximilian Seles, Georg C. Hutterer, Johannes Foßelteder, Marek Svoboda, Margit Resel, Dominik A. Barth, Renate Pichler, Thomas Bauernhofer, Richard E. Zigeuner, Karl Pummer, Ondrej Slaby, Christiane Klec , and Martin Pichler

Supplementary Information

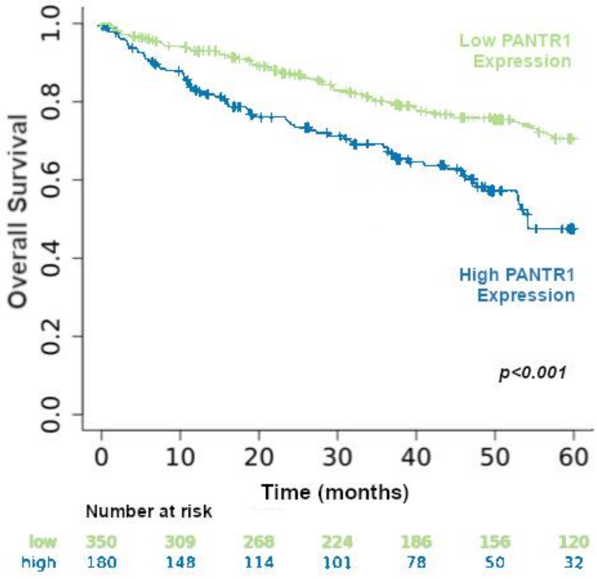

Figure S1. Kaplan-Meier plot comparing 5-year overall survival of clear-cell renal cell carcinoma (ccRCC) patients stratified by PANTR1 expression (low expression in green vs. high expression in blue, n = 530, hazard ratio: 2.19, 95% confidence interval: 1.59–3.03, $p = <0.001$). Data was derived from The Cancer Genome Atlas.

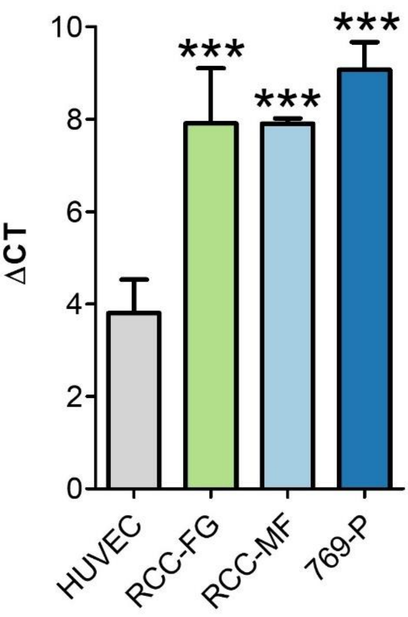

Figure S2. Relative PANTR1 expression was validated in human umbilical vein endothelial cells (HUVECs) and three RCC cell lines (RCC-FG, RCC-MF, 769-P) with qRT-PCR compared to the housekeeping genes using gene specific primers; *n* = 3.

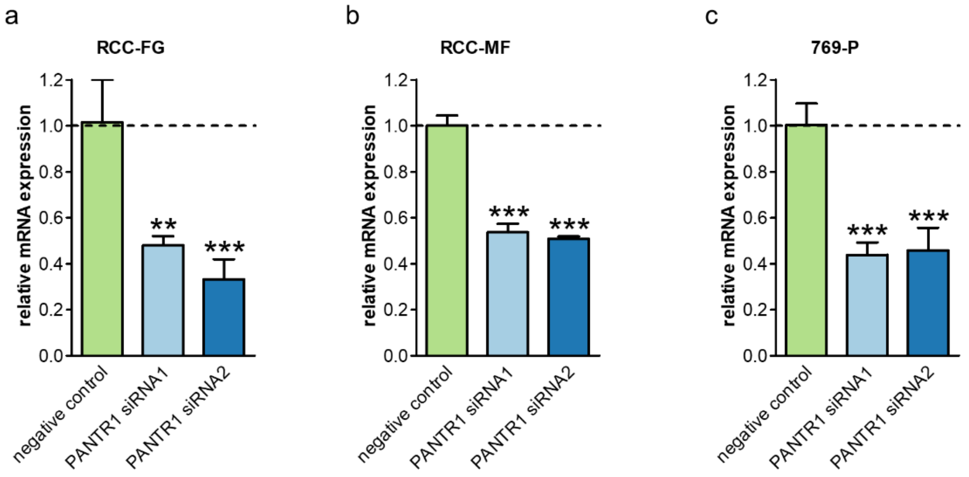

Figure S3. (**a**–**c**) Validation of siRNA-mediated PANTR1 knock-down efficiency in three RCC cell lines 48 h after transfection (RCC-FG, RCC-MF, 769-P) via qRT-PCR using gene specific primers; *n* = 3; mean±SD; $^{**}p < 0.01$, $^{***}p < 0.001$.

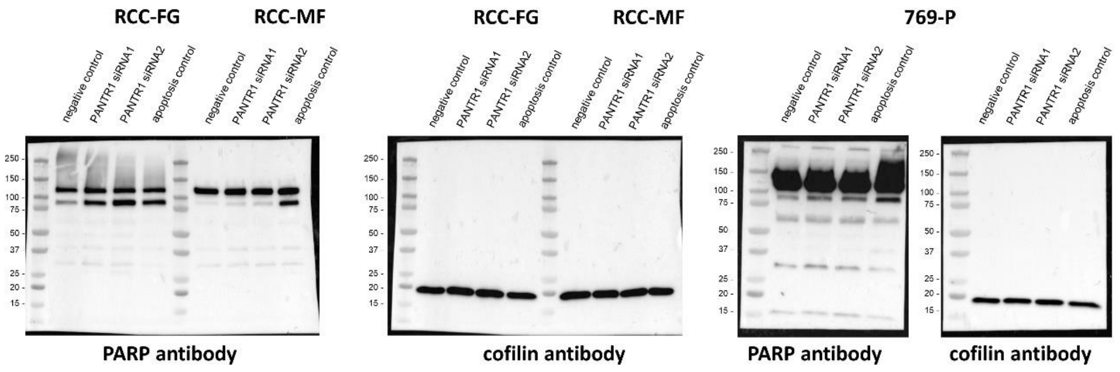

Figure S4. Uncropped images of Western Blots as shown in Figure 3.

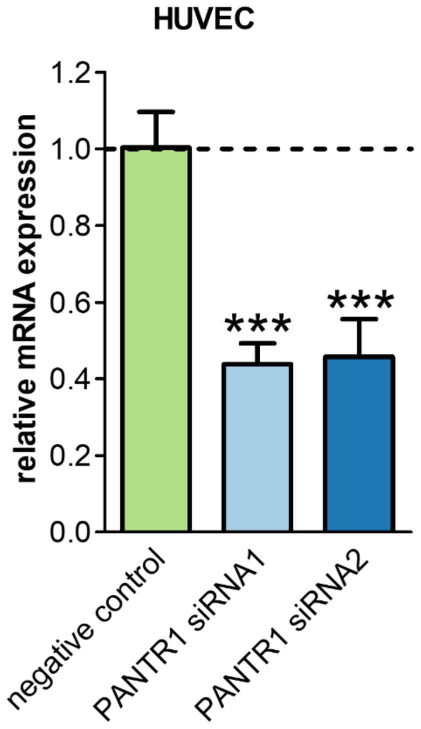

Figure S5. Validation of siRNA-mediated PANTR1 knock-down efficiency 48 h after transfection in HUVECs via qRT-PCR using gene specific primers; n = 3; mean±SD; ** $p < 0.01$, ***$p < 0.001$.

Supplementary Table 1. Sequences of primers used for qRT-PCR.

Gene	forward	reverse
**PANTR1**	CAT CAG GGG AGC AAC GTG AA	AGA GGA TGT GGT CAC TCC AGA
**LAMC2**	TGG ATG CAG TAC AGA TGG TGA TT	CCA GCC CCT CTT CAT CTA CAC
**VEGF-A**	CTC AGG GTT TCG GGA ACC AG	GTC GAT GGT GAT GGT GTG GT
**GAPDH**	AAG GTC GGA GTC AAC GGA TTT	ACC AGA GTT AAA AGC AGC CCT G
**U6**	CTC GCT TCG GCA GCA CA	AAC GCT TCA CGA ATT TGC GT

SUPPLEMENTARY TEXT 1

MIQE Guidelines for qPCR experiments

Experimental design

Definition of experimental and control groups:

- Transfected samples:
  - control group: cells transfected with scrambled negative control siRNA (50 nM, 48 h)
  - target group: cells transfected with siRNAs against PANTR1 (50 nM, 48 h)
    - PANTR1 siRNA1
    - PANTR1 siRNA2
- Untransfected samples: cDNA of HUVECs, RCC-FG, RCC-MF, 769P cells

Number within each group: at least 3 biological replicates

Sample

Description: Transfected cells were harvested 48 h after transfection and RNA was isolated. Untransfected cells were harvested at approx. 80–90% confluency and RNA was isolated.

Volume/mass of sample processed: Cells were grown in 6-well plates, each well was used for one sample.

Processing procedure: growth medium was removed from cells, cells were washed twice with PBS and resuspended in 1 mL Trizol per well.

If frozen, with what and how quickly? RNA was isolated directly after harvesting the cells.

Nucleic acid extraction

Procedure and instrumentation: RNA was isolated according to the principle of organic extraction.

Name of kit and details of any modifications: RNA was isolated using TRIzol reagent strictly following the protocol of the manufacturer (Invitrogen/Thermo Fisher).

Details of DNase or RNase treatment: all procedures were performed with RNase/DNase-free plastic ware, water and surfaces (surfaces, pipettes etc. were cleaned with RNase Zap spray).

Nucleic acid quantification: measurement with a spectrophotometer

Instrument and method: Nanodrop 2000, microvolume spectrophotometer without cuvettes, 1 µL sample on sample spot.

Purity (A_{260}/A_{280}): Purity of samples used in this study were between 1.85 and 2.02; samples with a purity lower than 1.75 were excluded.

RNA integrity: method/instrument: Measurement of A260, A280 and A230 for contaminations as well as gel electrophoresis to visualize RNA integrity.

Reverse transcription

Complete reactions + amount of RNA and reaction volume: 1 µg of RNA per sample is reverse transcribed to cDNA in a total volume of 20 µL. Master mix: 2 µL gDNA wipeout buffer, appropriate volume of RNA to achieve 1 µg, 4 µL RT buffer, 1 µL RT Primer Mix, 1 µL Reverse Transcriptase, appropriate volume of RNase free water to a total of 20 µL.

Reverse transcriptase and concentration: Mixture of the Qiagen products Omniscript Reverse Transcriptase and sensiscript Reverse Transcriptase at an optimal concentration to transcribe up to 1 µg of RNA (according to the manufacturer). RNase Inhibitor is already included.

Temperature and time: gDNA wipeout step: 2 min, 42 °C to eliminate genomic DNA contamination; reverse transcription runs at 42 °C for 60 min, followed by heat inactivation at 95 °C for 3 min.

Manufacturer of reagents and catalogue numbers: QuantiTect Reverse Transcription Kit (Qiagen), Cat No./ID: 205313

qPCR target information

Gene symbol (Entrez Gene ID): PANTR1, POU3F3 Adjacent Non-Coding Transcript 1 (100506421); LAMC2, Laminin Subunit Gamma 2 (3918); VEGF-A, Vascular Endothelial Growth Factor A (7422)

Sequence and accession number: PANTR1 for transcript variants 1-4: NR_037883.1, NR_131233.1, NR_131234.1 and NR_131235.1; LAMC2 for transcript variants 1-2: NM_005562.3 and NM_018891.3; VEGF-A for transcript variants 1-10: NM_ 001171623-30.1, NM_001204384.1 and NM_001287044.2.

Amplicon length: primers were designed with NCBI Primer blast, amplicon length was set between 70–150 bp.
In silico specificity (BLAST): All oligonucleotide sequences have been tested for specificity with NCBI Blast as well as sequence alignment via clustal omega.
Location of each primer by exon or intron: primers were designed exon-exon spanning, to avoid amplification of genomic DNA contaminations.
What splice variants are targeted?: Primers were designed to record all splice variants.
qPCR oligonucleotides
Primer sequences:
PANTR1_for: CAT CAG GGG AGC AAC GTG AA
PANTR1_rev: AGA GGA TGT GGT CAC TCC AGA
LAMC2_for: TGG ATG CAG TAC AGA TGG TGA TT
LAMC2_rev: CCA GCC CCT CTT CAT CTA CAC
VEGF-A_for: CTC AGG GTT TCG GGA ACC AG
VEGF-A_rev: GTC GAT GGT GAT GGT GTG GT
GAPDH_for: AAG GTC GGA GTC AAC GGA TTT
GAPDH_rev: ACC AGA GTT AAA AGC AGC CCT G
U6_for: CTC GCT TCG GCA GCA CA
U6_rev: AAC GCT TCA CGA ATT TGC GT
Location and identity of any modifications: None
Manufacturer of oligonucleotides: Eurofins genomics
Purification method: HPLC
qPCR protocol
Complete reaction conditions: 1 µg of RNA was reverse transcribed with QuantiTect Reverse Transcription kit (Qiagen, Hilden, Germany). qPCR was set up in 384-well format. Per reaction the composition of the master mix was the following: 5 µL QuantiTect SYBR Green Mastermix, 0.4 µL forward primer, 0.4 µL reverse primer (working stock concentration 10 µM) and 3.2 µL RNase-free water = total of 9 µL.
Reaction volume and amount of cDNA: 10 ng cDNA/reaction in a volume of 1 µL. Total reaction volume (master mix + cDNA) = 10 µL per 384-well.
Primer, Mg^{2+}, and dNTP concentrations: Primers are used at a final concentration of 0.5 µM, Mg^{2+} (which is already contained in the 2× SYBR Green Master mix) has a final concentration of 2.5 mM and dNTPs are already included in the 2× SYBR Green Master mix.
Polymerase identity and concentration: HotStartTaq DNA Polymerase originally isolated from *Thermus aquaticus* and cloned into *Escherichia coli.* According to the manufacturer the optimal concentration of Polymerase is present in the master mix.
Buffer/kit identity and manufacturer: QuantiTect SYBR Green PCR Kit (Qiagen), Cat No./ID: 204145
Additives (SYBR Green I, DMSO): We use the kit components as suggested by the manufacturer and do not add any additives.
Complete thermocycling parameters:

- PCR initial activation: 15 min, 95°C, ramp rate: 4.4 °C/s (1 cycle, analysis method: none)
- Amplification (40 cycles, recorded by instrument: 35 cycles, analysis method: quantification):
    - Denaturation: 15 s, 94°C, ramp rate: 2.2 °C/s
    - Annealing: 30 s, 55°C, ramp rate: 2.2 °C/s
    - Extension: 30 s, 70°C, ramp rate: 4.4 °C/s
- High Resolution Melting (1 cycle, analysis method: melting curve)
    - 5 s, 95°C, ramp rate: 4.4 °C/s
    - 1 min, 65°C, ramp rate: 2.2 °C/s
- Cooling (1 cycle, analysis method: none)
    - 10 s, 40°C, ramp rate: 2.2 °C/s

Manufacturer of qPCR instrument: Roche LightCycler 480

qPCR validation

Specificity: Melting temperature analysis has been performed for all primers.

For SYBR Green I, C_q of the NTC: We always run NTC and RT- samples as negative controls, only values in the not detectable range (with our settings Cq of 35) are acceptable.

PCR efficiency calculated from slope: $E = -1 + 10^{(-1/slope)}$; it ranged between 94–105%.

r^2 of calibration curve: between 0.96–1.0

Data analysis

qPCR analysis program: for analysis of qPCR data Excel has been used

Method of Cq determination: 2^{-ddCT} method has been used.

Outlier identification and disposition: Due to the setup of our study, we did not have outliers.

Results of NTCs: Only NTC values in the not detectable range have been considered as acceptable.

Justification of number and choice of reference genes: We have used two reference genes. They are well established in our lab, are the standard reference genes used and have been stable throughout all our experiments.

Description of normalization method: siRNA-Transfected samples have been normalized to negative control siRNA samples. For evaluation of PANTR1 expression among cell lines, PANTR1 mRNA levels have been normalized to housekeeping gene levels.

Number of biological replicates: at least 3 individual biological replicates per condition.

Number of technical replicates: on 384-well plate samples have been pipetted in duplicates.

Repeatability: We have analyzed samples on different 384-well plates in order to assure reproducibility of results.

Statistical methods for results significance: Since we have at least two experimental conditions (two different siRNAs against PANTR1) we have used one-way ANOVA and Bonferroni Posthoc test.

Software (source, version): Statistics were calculated with GraphPad Prism V5.01.
